# Realistic and critical review of the state of systemic antimicrobial peptides

**DOI:** 10.5599/admet.1215

**Published:** 2022-01-20

**Authors:** Guangshun Wang, Abraham Fikru Mechesso

**Affiliations:** Department of Pathology and Microbiology, College of Medicine, University of Nebraska Medical Center, 985900 Nebraska Medical Center, Omaha, NE 68198-5900, USA

**Keywords:** Antibiotics, antimicrobial peptides, peptide design, MIC, PK, PD, systemic efficacy, toxicity, intravenous injection

## Abstract

Antimicrobial peptide research remains active not only because of the growing antibiotic resistance problem but also our desire to understand the role of innate immune peptides in host defense. While numerous peptides are currently under active development for topical use, this article highlights peptides with systemic efficacy. The scaffolds of these peptides range from linear to cyclic forms. The neutropenic mouse model is well established to illustrate antimicrobial efficacy from direct killing. The majority of tests, however, are conducted using normal mice so that both direct antimicrobial and immune regulatory effects can be characterized. These systemic examples underscore the possibility of adding new candidates to the list of the existing peptide antibiotics to more effectively combat antibiotic-resistant bacteria, fungi, and parasites.

## 1. Introduction

The current SARS-CoV-2 pandemic is a reminder to human beings that pathogenic microbes can have a tremendous impact on every aspect of our society. In addition to viruses, antibiotic-resistant bacteria, fungi and parasites constitute a new potential threat. It is projected that 10 million people could die from untreatable infections by 2050 [1]. Consequently, it becomes urgent to search for novel antimicrobials.

Innate immune antimicrobial peptides (AMPs) play a critical role in protecting the hosts from infection [2,3]. Their lasting efficacy rejuvenates our interest in developing them into novel antibiotics. Indeed, some peptide antibiotics such as gramicidin, vancomycin, daptomycin and colistin are already in clinical use [4]. According to the updated antimicrobial peptide database (*https://aps.unmc.edu*), over 3000 such peptides have been discovered and characterized from natural sources, ranging from bacteria to animals [5,6]. Many AMPs are cationic and possess broad-spectrum antimicrobial activity against both Gram-positive and Gram-negative bacteria. Some peptides, however, eliminate only Gram-positive or Gram-negative bacteria. Frequently, AMPs consist of both basic and hydrophobic amino acids. While basic amino acids initiate anionic bacterial recognition, hydrophobic amino acids are important for subsequent peptide anchoring into membranes. This is accompanied by a structural transition from random coils to an amphipathic α-helical structure [2,3,7]. In addition to the α family, there are three other types of structural families: β, αβ, and non-αβ [8]. Representative structures for these four families can be viewed on the face page of the AMP database website above. Some AMPs have a folded structure even in an aqueous solution. One molecular mechanism results from the forced folding of disulfide bonds. As a consequence, α-defensins are known to adopt β-sheet structures. Beta-defensins, longer than α-defensins, can incorporate an α-helical element to generate a mixed αβ fold. Like helical peptides, defensins have an effect on membranes as well. Some defensins, however, work by inhibiting cell wall synthesis [9,10]. There are also AMPs that form neither a regular α-helix nor a β-sheet structure. Proline-rich peptides are such examples that inhibit bacterial growth by binding to ribosomes [11]. The structural diversity of AMPs offers numerous R&D opportunities for developing novel peptide antibiotics.

This minireview highlights AMPs with the potential to treat systemic infections. We discuss peptide toxicity, in vivo efficacy, and pharmacokinetic (PK) and pharmacodynamic (PD) studies. These examples support the potential of engineered AMPs as novel systemic antibiotics.

## 2. Peptide design: antimicrobial robustness, selectivity and stability

The basic goal of peptide design is to identify potent peptides with minimal or no toxic effects on normal cells. It is evident that basic and hydrophobic amino acids are the two key elements in designing amphipathic antimicrobial peptides [7,12,13]. Our current knowledge implies that basic amino acids are key to the inhibition and killing of Gram-negative bacteria, whereas hydrophobic amino acids are critical to eliminating Gram-positive bacteria such as methicillin-resistant *Staphylococcus aureus* (MRSA) [14]. Although reducing peptide hydrophobicity is a general strategy to improve peptide cell selectivity [15], basic amino acids also play a role [16]. Hence, the finally designed peptide achieves an optimal combination of hydrophobic and basic amino acids. Selected peptides discussed in this review are listed in Table 1. These peptides were obtained by modifying natural peptides or designed based on the key elements of AMPs. A3-APO, Onc72, Onc112, and Api137 are derived from proline-rich peptides originally discovered in insects [17-19]. Gomesin is a short β-hairpin peptide isolated from scorpions [20]. AMPR-11 is a peptide derived from mitochondrial non-selective channel Romo-1 [21]. C10-KR8d is a lipopeptide designed based on the minimal antibacterial peptide KR-12 of human cathelicidin LL-37 [22], whereas OH-CATH30 is a truncated fragment of the king cobra cathelicidin OH-CATH [23]. Some peptides are de novo designed. WLBU2 is a synthetic peptide designed using valine, tryptophan, and arginine [24]. DFT503 and DFT561 [25] are derivatives of the DFTamP1, which is the first database filtering technology designed peptide obtained by extracting the key parameters (peptide length, charge, hydrophobicity, and structure) from the antimicrobial peptide database [26]. Horine and verine were designed by combining database filtering with structural knowledge [27].

Another important goal of peptide design is to improve peptide stability to proteases. Several strategies are utilized to enhance peptide stability: terminal capping, incorporation of non-standard amino acids, and peptide cyclization [28]. For example, the incorporation of D-amino acids increased the half-life (t_1/2_) of horine from ~10 min (L-form) to 60 min (D-form) in vivo [27]. In the case of C10-KR8, the D-form is preferred over the L-form since the D-from did not bind as many serum components as the L-form based on mass spectrometric studies [22]. There are also other methods to improve peptide stability. While peptide mimicries may be made to eliminate susceptible peptide bonds entirely [29-31], formulations can protect peptides from protease degradation [32,33].

Subsequently, after peptide optimization, it is necessary to test peptide antimicrobial robustness. This is because AMPs such as LL-37 are known to lose activity in the presence of physiological salts, serum, and under acidic conditions [22,24,34]. It is anticipated that the peptides are more likely to display in vivo efficacy if they can retain antimicrobial activity under such conditions. To identify candidates with such properties, we assembled a pipeline of in vitro filters by measuring peptide MIC values in rich media supplemented with salts, serum, and at an acidic pH. We found that very few peptides retain antimicrobial activity under all these conditions. It was encouraging that our database-designed peptides that crossed these barriers did show systemic efficacy in vivo [22,27].

## 3. In vivo toxicity of designed antimicrobial peptides

Nephrotoxicity of colistin is the major side effect of this antibiotic [35,36]. Therefore, there is a desire to develop the next generation of peptide antibiotics with minimized toxicity. To evaluate the peptide toxicity in vivo, the commonly used routes for peptide administration are intraperitoneal (i.p.), intravenous (i.v.), intramuscular (i.m.), and subcutaneous (s.c.) injections (Table 1). Of significant interest is the peptide toxicity after intravenous injection. For A3-APO, mice survived with severe transient side effects at 25 mg/kg (i.p.), but a single dose of 50 mg/kg was lethal, indicating a maximum tolerated dose (MTD) was 20 mg/kg. It is equal to the No Observed Adverse Effect Limit (NOAEL) in this case. In the same model, the 50 % lethal dose (LD_50_) of colistin was found to be less than 40 mg/kg [17]. The LD_50_ of OH-CATH30 after i.p. injection was 120 mg/kg and mice survived when subcutaneously injected at 160 mg/kg [23]. The WLBU2 peptide was found to have an MTD of 12 mg/kg (i.v.) [24]. This is fourfold higher than the treatment dose at 3 mg/kg. Four alternating D,L-cyclic peptides (6752, 6756, 6853 and 7251) displayed MTDs in the range of 20-25 mg/kg or above depending on the amino acid sequence [37]. These concentrations allowed a dose-dependent treatment at 16 mg/kg or below. Likewise, teixobactin showed no sign of toxicity when administered intravenously at a single dose of 20 mg/kg [38]. A single i.v. injection of AMPR-11 (100 mg/kg), tenfold higher than the treatment dose into mice, did not show severe clinical signs of toxicity for 15 days [21]. After one week of daily i.p. injection at the treatment dose (10 mg/kg), horine showed no side effects on body weight, animal behavior, or kidney histology [27]. Although a slight body weight loss (12.1 %) was noted after daily i.v. injection (10 mg/kg) for a week, we did not observe any other side effects. These results are encouraging and will lead to the discovery of novel peptides with further reduced toxicity and enhanced potency.

## 4. Systemic efficacy of antimicrobial peptides

While most candidates are developed for topical use, some laboratories have observed systemic efficacy for designed peptides (Table 1). Both neutropenic and normal mice are utilized for systemic efficacy evaluation. Neutropenic mice are a classic model to establish direct antimicrobial efficacy and for studying PK/PD of antibiotics since the immune effects are suppressed [39,40]. It is important to establish that bacteria had spread to different organs prior to antimicrobial treatment. After i.p. injection, Szabo and colleagues [17] found a 30-minute delay was sufficient for *E. coli* to spread all over the body of mice. Mor and colleagues observed the spread of *Escherichia coli* in neutropenic mice one-hour post i.p. infection [31]. We also detected MRSA or *Klebsiella pneumoniae* in different murine organs two hours post-infection [25,27]. Selsted and colleagues also found the dissemination of *K. pneumoniae* to various organs one-hour post i.p. infection [41]. The peptide efficacy is often reflected by a dose-dependent colony-forming unit (CFU) drop as well as the survival advantage of mice in the treated group relative to the untreated infected group. Using the neutropenic thigh model, Dartois et al. [37] made alternating D,L-cyclic peptides and observed a decrease of the *S. aureus* load by 2.1-3.0 log units after i.v. treatment (8 mg/kg) two hours post i.m. infection. Although effective in vitro, two such peptides (1316 and 1150) failed to show in vivo activity, probably due to low bioavailability. Using the same model, Li et al. [23] tested the efficacy of OH-CATH30 immediately after thigh infection at 1, 10, and 20 mg/kg via a single s.c. treatment. In the protection experiment, the survival rate of mice decreased from 100 % to 57-70 % when the treatment time was delayed from one hour to four hours post i.p. infection (*E. coli* at 10^8^ CFU). Using CD-1 neutropenic mice infected with MRSA intraperitoneally, teixobactin (MIC 0.25 μg/ml against MRSA) shows dose-dependent efficacy (i.e., CFU decrease) after a single dose i.v. treatment two hours post-infection [38].

Mor observed systemic efficacy of the OAK peptide mimics in neutropenic mice but not two amphibian peptide derivatives [31]. Likewise, we did not observe systemic efficacy in this model for a human cathelicidin LL-37 derived peptide [25]. One possibility is that the peptide associates with other molecules in mice and is not available to attack bacteria. Our reasoning is in line with the molecular promiscuity of LL-37 (binding to numerous molecular partners such as human serum proteins and nucleic acids) [22,24,34]. Similarly, indolicidin did not show a protective effect when treated in the free form. After formulation, however, it protected mice by 30 % [32]. These results indicate that not all AMPs are able to display systemic efficacy in mice in the free form. Taking this into consideration, we then aimed at identifying more robust peptides that retain activity under various conditions by expanding the database filtering technology from in silico to in vitro. Our database-designed peptides are particularly effective against Gram-positive pathogens such as MRSA and vancomycin-resistant *Enterococci* (VRE). In addition, we discovered that an increase in cationic amino acids led to an increase in toxicity and a loss of in vivo activity of the DFT-designed peptides in neutropenic mice [25]. In keeping with this low cationicity idea, we have designed even shorter peptides such as horine and verine to reduce production costs [27]. These two peptides share a similar amino acid composition but possess different amino acid sequences. They also have different 3D structures and activity spectra: horine mainly against Gram-positive MRSA and verine against both Gram-positive and negative pathogens. During a five-day observation, a single dose injection of horine at 10 mg/kg (i.p.) protects 87.5 % mice infected with MRSA identical to vancomycin, whereas 81.8 % mice infected with antibiotic-resistant *K. pneumoniae* E406-17 survived after a single dose of verine treatment (15 mg/kg) 2 h post infection. This protective effect of verine was better than doripenem (50 % protection) at the same dose. Likewise, we observed a significant decrease in bacterial burden (1-4 logs) in a variety of murine organs, including the spleen, lung, kidney, and liver, when treated either intraperitoneally or intravenously [27]. These results document direct antimicrobial efficacy for AMPs.

Other laboratories have primarily used normal (non-neutropenic) mice to test peptide efficacy. In such a model, the peptide may achieve efficacy by direct killing and/or immune boosting. Usually, a high bacterial inoculum is required for infection (e.g., ~10^8^ CFU for MRSA and ~10^7^ CFU for *P. aeruginosa*). Deslouches et al. [24] observed a systemic effect for WLBU2 treated i.v. at 3 mg/kg 30 to 45 min after *P. aeruginosa* i.p. infection. No bacteria were recovered after 7 to 10 days from tissues or blood. Several peptides derived from proline-rich AMPs show systemic efficacy in mice infected intraperitoneally. Examples include A3-APO, Onc72, Onc112, and Api137 (Table 1). The designer peptide, A3-APO, is effective in multiple models to protect mice from *E. coli* Neumann infection in a dose-dependent manner. At 10 mg/kg, it was as efficacious as imipenem in the long term. However, s.c. treatment was found to be ineffective against *E. coli*. The i.p. efficacy is proposed to result from both direct killing and immune-stimulatory effects [17]. Onc72 and Onc112 (2.5 or 5 mg/kg injected i.p. at 1, 4, and 8 h post-infection) were able to protect mice in a septicaemia model due to *E. coli* ATCC 25922 infection [18]. Api137 (an apidaecin derived peptide) showed a dose-dependent protection of the CD-1 mice (67 % survival over 5 days) from *E. coli* ATCC 25922 infection when treated subcutaneously but not intravenously [42]. Although the minimal bactericidal concentrations (MBC) of AMPR-11 are moderate (~50 μM), it increases mouse survival rate by 60 % after a single dose (10 mg/kg, i.v.) treatment one-hour post-infection with *S. aureus, Pseudomonas aeruginosa, K. pneumoniae*, or *Acinetobacter baumannii* [21]. This efficacy is comparable to that achieved by imipenem injected four times (every 12 h, i.p.) 24 h post-infection. It is proposed that the observed efficacy results from multiple factors, including rapid killing, a lack of binding to serum/lipoproteins, and lipopolysaccharides (LPS) neutralization. Although not discussed, peptide-induced immune stimulation as evidenced by LPS neutralization could also be an important factor. Zhang et al. [43] observed a 2-3 log CFU decrease (relative to PBS treated) in mice injected with DP7 (2 mg/kg, i.v.) one-hour post i.v. infection similar to vancomycin at 10 mg/kg. The RTD-1 peptide, a theta-defensin, shows systemic efficacy against intravenously infected *Candida albicans* when treated i.p. daily [44]. Interestingly, the treatment efficacy of RTD-1 at 5 mg/kg does not depend on administration routes. However, a further delay of treatment from 1, 3, to 6 h after infection reduces peptide efficacy. The fact that the measured peptide concentrations in mice are 5-25 fold lower than MICs (>100 μg/mL) against *C. albicans* in the presence of 50 % serum led to the conclusion that peptide efficacy did not result from direct antifungal effect. The same conclusion has been arrived in a recent study using MTD12813, an improved version of RTD-1, to treat mice (i.p.) infected with Gram-negative *E. coli* or *K. pneumoniae* (i.p.) [41]. Likewise, Rossi et al. [20] demonstrated the impact of gomesin (a scorpion peptide) on the host immune response against *Candida* infection in mice. In addition to bacterial killing [23], Li et al. [45] showed that OH-CATH30, a snake antimicrobial peptide, also induces an innate immune response to help the host combat bacterial infection. These examples indicate that AMPs can selectively boost the host immune response by regulating the release of proinflammatory cytokines and chemokines and promoting the chemotaxis of immune cells [45,46].

In summary, these examples underscore the feasibility of identifying systemic AMPs. The systemic efficacy of these peptides could result from direct antimicrobial killing, immune stimulation, or a combination of the two mechanisms. In addition, in vivo efficacy could depend on experimental conditions. For instance, cyclic peptides were effective when administered intravenously but not orally or subcutaneously [37]. If we define the selectivity index as the ratio between the MTD and peptide treatment dose, there is a therapeutic window of up to 10 for these peptides to treat bloodstream infections (Table 1) and up to 100 to treat animals intraperitoneally [41].

## 5. Peptide pharmacokinetics and pharmacodynamics

The PK and PD data for antimicrobial peptides are very limited. Dartois and colleagues [37] followed the serum concentrations of peptides 6752 and 6853 after treatment at different doses. Maximum concentration (*C*_max_) and the area under the concentration-time curve (AUC) increased steadily after injection from 2 to 8 mg/kg. In contrast, peptides 1316 and 1150, which did not display in vivo efficacy, showed significantly lower *C*_max_ values, indicating the need for a sufficient amount of peptide (e.g., above MIC) for in vivo bacterial elimination. The *t*_1/2_ values for these cyclic peptides ranged from 1.2 to 3.9 h. Consistent with in vitro results, the in vivo anti-MRSA effect of the peptide did not depend on the susceptibility of bacteria to conventional antibiotics. The faster killing by peptide 6752 (membrane disruption) than vancomycin and oxacillin (cell wall inhibition) in vivo is consistent with their mechanisms of action. Interestingly, when treated at 16 mg/kg in the thigh model, the prolonged antibacterial activity (PAE) was greater than 6 h for vancomycin and >7 h for 6752. A significant amount of the peptide was cleared renally. Ling et al. [38] showed that intravenous injection of a single dose of teixobactin (20 mg/kg) resulted in serum concentrations above the MIC for 4 h, explaining the peptide efficacy in vivo. Schmidt et al. [19] also showed that when Api88 or Api137 was injected i.v. or i.p. at doses of 5 and 20 mg/kg, their plasma levels were similarly low (<3 μg/mL). Comparable levels of these peptides accompanied by the same major metabolites were detected in blood, urine, kidney, and liver homogenates. Api137 was rapidly degraded at the C-terminus, while Api88 was rather stable. The high efficacy in murine infection models and the rapid clearance (60-90 min) of these peptides indicate that their in vivo efficacy is also related to the *C*_max_. Rossi et al. [20] found that gomesin mainly accumulated in the liver and rapidly eliminated from the circulation. However, the excretion route remained unexplored. In our recent study [27], we observed different half lives for the L- and D-forms of horine (*t*_1/2_ ~10 min and ~1 h) in murine blood after i.v. injection and a decrease in the amount of the peptide in blood is accompanied by an increase in other organs, including the spleen, lung, kidney, and liver. While the plasma level of D-horine remains above the MIC (4 μM) for about one hour, the L-form of horine is rapidly degraded. The fact that both peptides demonstrate in vivo efficacy indicates rapid bacterial killing by horine, consistent with in vitro findings [27].

## 6. Concluding remarks and future directions

There is a clear need for systemic antimicrobials to treat bloodstream infections caused by antibiotic-resistant pathogens, such as bacteria, fungi, and parasites. Our establishment of a pipeline of peptide filters from in vitro to in vivo, inspired by our in silico filtering technology, presents a useful strategy for identifying additional systemic candidates to treat bloodstream infections [25]. In several cases, evidence has been obtained for the establishment of systemic bacterial infections before treatment. Based on limited systemic examples summarized here, we conclude that not all AMPs are able to display systemic efficacy in vivo. Some notable features for these systemic peptides in Table 1 are (1) low hydrophobic Pro-rich peptides, (2) low cationic database-derived peptides, and (3) cyclic peptides. Also, a small size is preferred to reduce production costs. It is proposed that a compact structure can reduce potential binding to other molecules. Such features might have improved the bioavailability of these peptides in vivo. In addition, the systemic effect of a peptide may depend on the type of animal models, bacteria species, bacterial inoculum, treatment time post-infection, peptide dose, and treatment frequency. It is clear in each case that a sufficient amount of antimicrobials is required to achieve in vivo efficacy. Therefore, further studies are required:

1. To identify the in vitro conditions useful for identifying systemic peptides;
2. To evaluate peptide toxicity more completely by using a variety of mammalian cell lines and animal models; and
3. To characterize the PK/PD properties of the identified AMP candidates since only limited data are available at present.

Finally, one can also learn from the peptide antibiotics already in clinical use (Table 2). These include vancomycin, daptomycin, colistin, and their derivatives recently approved by FDA. These peptide-based antibiotics are characterized by high efficacy and selectivity, low toxicity, low metabolic stability, and rapid renal clearance [42]. Notably, most known peptide antibiotics are cyclic and differ from the classic amphipathic helical peptides. Hence, we anticipate more attention to cyclic peptides in developing a new generation of future antibiotics. We also anticipate a continued search of novel peptide structures in nature by combining genetic, bioinformatic, proteomic, and structural biology approaches. It is proposed that a combination of AMPs with existing antibiotics can bring forth additional treatment benefits otherwise unachievable [4,47]. An improved understanding of AMPs in the host as well as in association with microbiota will contribute to future personal and precise medicine.

## Figures and Tables

**Table 1.** Antimicrobial peptides with systemic efficacy

Peptide	Mice	Microorganism	Infection route	Treatment route	Bacterial CFU drop	Survival rate	PK/PD	Ref (Year)
Indolicidin liposome	BALB/c	*Aspergillus fumigatus* spore	IV	IV	-	30% of infected mice survived after treatment at 40 mg/kg	MTD 40 mg/kg	[32] (1995)
P19(8)	Mice	*Candida albicans*	IV	IV	-	A modest prolongation in survival time of infected mice after treatment with 5 mg/kg of the peptide	25 mg/kg	[48] (2002)
BMAP-28	BALB/c	*Staphylococcus aureus*	IV	IV	~3 log	75% of infected mice survived after BMAP-28 treatment	ND	[47] (2004)
Cyclic 6752	Mice	*S. aureus* (MSSA/MRSA)	IM	IV	2.9-3.6 log	ND	-The decline in concentration was best described by a two-compartment model. -Most of the injected compounds are cleared renally. -The Cl, T1/2β and Vss were 0.19-0.26 L/Kg/hr, 1.2-2.2 hr, and 0.33-0.49 Lit/kg, respectively.	[37] (2005)
WLBU2	Swiss Webster	*Pseudomonas aeruginosa* PAO1	IP	IV	No bacteria etected after 7 to 10 days post treatment	ND	MTD = 12 mg/kg	[24] (2005)
Ranalexin and recombinant lysostaphin	Mice	MRSA	IV	IV	1 log	ND	No activity in serum	[49] (2010)
OAKs C_12(ω7)_K-β_12_	Mice	*S. aureus*	IP/IM	IP/IM /IV	~3 log	30% survival at 2 mg/kg and 90% at 5 mg/kg.	After SC administration, remained stable in the circulation for >1 h	[50] (2010)
A3-APO dimer	CFW-1	*Eschersichia coli*	IP	IP	3-4 log in both cisplastin pre-treated and non-–treated mice.	80-100% survival in cisplastin non-treated mice, 40-100% in cisplastin-treated mice.	MTD 25-50 mg/kg	[17] (2010)
A3-APO	CD-1	*Acinetobacter baumannii*	IP	IM/IV	0.5-2 log	100% survived after 12 h and only 14.3% survived after 24 h.	The IM therapeutic index of the peptide (>12) appeared to be higher than that of colistin (around 5–6); MTD>60 mg/kg.	[51] (2010)
RP-1	BALB/c	*L. infantum chagasi*	IV	IV	Significant parasite load drop in liver	ND	Retained activity against the parasite in blood	[52] (2012)
Gomesin	BALB/c	*Candida albicans*	IV	IP	0.5-1 log	All died on the 18^th^ day of treatment, three days later than the PBS control	Mainly distributed in liver (60%) for up to 24 h post-injection.	[20] (2012)
OH-CATH30	Mice	*E. coli* *P. aeruginosa* *S. aureus*	IP	IP	4-5 log	The survival rate: 60 to 90%	*t*_1/2_ < 60 min.	[23] (2012)
Teixobactin	CD-1	MRSA	IP	IV	~4 log/thigh	In a septicemia protection model, 100% survival during 48 h when treated above 0.5 mg/kg.	Concentration above the MIC for 4 h after a single dose of 40 mg/kg; t_1/2_, 4.7 h; AUC to last, 57.8 μg-h/mL; Volume of distribution, 47 mL; MTD > 20 mg/kg.	[38] (2015)
Onc112 Onc72	Mice	*E. coli*	IP	IP	4-5 log 10 unit	ND	Onc72: t_1/2_, 12 min; volume of distribution, 32 mL; Onc112: t_1/2_, 19.3 min; volume of distribution, 13 mL.	[18] (2016)
DP7-liposome	Mice	MRSA	IP	IV	~1 log 10 unit	ND	ND	[53] (2016)
Api137	Mice	*E. coli*	IP	IP	ND	100%	Detected in blood, urine, and kidney, and liver homogenates at similar levels, with rapid clearance within ~90 min.	
Chex1-Arg20 amide Chex1-Arg20 hydrazide	Mice	*A. baumannii*	IP	IM	~0.5-1.5 log 10 unit	Majority of the amide analog treated mice survived	MTD: 10 mg/kg for hydrazide analog and 25 mg/kg for amide analog.	[54] (2018)
RTD-1	CD-1	*C*. *albicans*	IV	IV/SC/IP	~1-3 log fold	A single dose of RTD-1 significantly enhanced survival regardless of the routes of administration	MTD > 50 mg/kg; a two-compartment model best described the IV data. Slowly absorbed IP with 63.4% bioavailability.	[44] (2019)
Api137	NMRI	*E. coli*	IP	IV/SC	ND	Survival 67%/IP and 33%/IV and SC administration	*C*max 23 mg/L (single IV 20 mg/kg); Low plasma levels, high volumes of distribution and low serum stability. Rapidly eliminated from the blood stream.	[42] (2019)
DFT503	C57BL/6	MRSA USA300	IP	IP	1.5-1.8 log	ND	ND	[25] (2019)
DFT561	C57BL/6	MRSA USA300	IP	IP	0.8-1.8 log	ND	ND	[25] (2019)
Tachyplesin III	BALB/c	*Co-infection P. aeruginosa* and *A. baumannii*	IN	IV		Survival 60%	ND	[55] (2019)
DP7	C57BL/6	MRSA	IV	IV	~1.5-2.5 log	70-90% reductions in the lethality of infection		[43] (2019)
AMPR-11	C57BL/6	*S. aureus P. aeruginosa*, *K. pneumoniae*, and *A. baumannii*	IV	IV	~0.5–1 log	Survival >60%.	MTD 100 mg/kg; activity decreased in plasma/serum. t_1/2_, ~50 min in plasma.	[21] (2020)
Horine	C57BL/6 BALB/c	MRSA USA300	IP	IP/ IV	up to 4.5 log/IP; ~2-2.5 log/IV	87.5% survived/IP (cf. 87.5% vancomycin)	*t*_1/2_ for horine < 15 min and for D-horine ~1 h. D-form detected in lung, spleen, liver, and kidney in 24 h.	[27] (2020)
Verine	C57BL/6 BALB/c	*K. pneumoniae*	IP	IP/ IV	1-3 log/IP; up to 7 log/IV	81.8%/IP (cf. 50% doripenem)	ND	[27] (2020)
C10-KR8d	C57BL/6 BALB/c	MRSA USA300	IP	IP	~0.5–1 log	ND	MTD >20 mg/kg; D-form binds less to serum than L-form.	[22] (2021)

Abbreviations: MTD, Maximum tolerable dose; ND, Not determined; IP, Intraperitoneal; IV, Intravenous; SC, Subcutaneous; IN, intranasal

**Table 2.** Clinically used peptide antibiotics for systemic and skin infections^1^

Peptide antibiotic	Trade name	Class	Activity spectrum	MOA	Treatment	Route	Approved
**Colistin (polymyxin E)**	C*olistimethate* sodium, Coly Mycin M	Lipopeptide	G-	Membrane lysis	Multi drug- resistant G- infections	IV	1970
**Polymyxin B**	Poly-Rx	Lipopeptide	G-	Membrane lysis	Urinary tract and bloodstream infections	IV	1994
**Vancomycin**	Vancocin, Vancoled	Glycopeptide	G+	Inhibits cell wall synthesis	G+ infections	IV	1958
**Teicoplanin**	Targocid	Glycopeptide	G+	Inhibits cell wall synthesis	G+ infections	IM,IV	1980 Europe
**Telavancin (TD-6424)**	Vibativ	Lipoglycopeptide (vancomycin derived)	G+	Membrane lysis and inhibits cell wall synthesis	Skin infections (CSSSI)	IV	2009
**Oritavancin (LY 333328)**	Orbactive	(vancomycin-derived)	G+	Membrane lysis and inhibits cell wall synthesis	Skin infections	IV	2014
**Dalbavancin (BI 397)**	Dalvance; Xydalba	Lipoglycopeptide (teicoplanin analog)	G+ (MRSA)	Inhibits cell wall synthesis	Skin infections (best drug tolerability)	IV	2014; 2021
**Daptomycin**	Cubicin	Lipopeptide	G+	Membrane lysis	Skin infections	IV	2003

^1^G+, gram-positive bacteria; G-, gram-negative bacteria; IV, intravenous; IM, intramuscular; CSSSIs, complicated skin and skin structure infections
